# High-fat diet-induced obesity and insulin resistance are characterized by differential beta oscillatory signaling of the limbic cortico-basal ganglia loop

**DOI:** 10.1038/s41598-017-15872-x

**Published:** 2017-11-14

**Authors:** Lukas Maurer, Hui Tang, Jens K. Haumesser, Jennifer Altschüler, Andrea A. Kühn, Joachim Spranger, Christoph van Riesen

**Affiliations:** 1 Charité University Medicine Berlin, Department of Endocrinology and Metabolism, Berlin, Germany; 2 Center for Cardiovascular Research – Charité-Universitätsmedizin Berlin, Berlin, Germany; 3 DZHK (German Centre for Cardiovascular Research), partner site Berlin, Berlin, Germany; 4 Charité University Medicine Berlin, Department of Neurology, Movement Disorder and Neuromodulation Unit, Berlin, Germany; 5 Berlin Institute of Health (BIH), Berlin, Germany

## Abstract

The concept of brain circuit disorders has been proposed for a variety of neuropsychiatric diseases, characterized by pathological disturbances of neuronal networks including changes in oscillatory signaling of re-entrant cortico-subcortical loops in the basal ganglia system. Parts of this circuitry play a pivotal role in energy homeostasis. We therefore investigated whether high-fat diet (HFD) induced obesity is associated with changes in oscillatory signaling in the limbic cortico-basal ganglia loop. We performed multi-site *in-vivo* electrophysiological recordings of local field potentials within this network under urethane anesthesia in adult rats after 4 weeks of HFD feeding compared to age-matched controls. Recordings were performed at baseline and during systemic glucose challenge. Our analysis demonstrates increased oscillatory beta power in the nucleus accumbens (NAC) associated with decreased beta coherence between cortex and NAC in animals fed a HFD. Spontaneous beta oscillatory power strongly correlated with endocrine markers of obesity. The glucose challenge increased beta oscillations in control animals but not in animals receiving the HFD. Furthermore direct intracerebroventricular insulin injection increased beta oscillations in the NAC. The present study provides evidence for aberrant oscillatory signaling in the limbic cortico-basal ganglia loop that might contribute to the dysfunctional information processing in obesity.

## Introduction

The prevalence of obesity and type 2 diabetes (T2DM) has reached epidemic proportions worldwide^1^. Primarily reward related overconsumption of highly palatable, energy dense foods beyond homeostatic needs is considered a central aspect in the multifactorial pathogenesis of obesity and its accompanying metabolic distortions^2^. Dysfunctional information flow of cortico-striatal networks involved in metabolic regulation as well as reward processing has been suggested to be of importance for the pathophysiology of obesity^3,4^. The limbic cortico-basal ganglia loop plays a central role in the brains reward circuitry that is responsible for the processing of reward related aspects of food consumption and it is intricately interconnected with hypothalamic midbrain areas that regulate the organism’s homeostatic functions^5^. In addition, peripheral hormones like insulin, leptin and ghrelin have been shown to modulate neuronal function within the basal ganglia^6^. In the context of obesity, multiple functional brain imaging studies have demonstrated cortical and striatal areas show profound differences between lean and obese individuals^7,8^. In obese participants an enhanced activation during food anticipation accompanied by a decreased activation during food reward receipt was reported^9^. It has been hypothesized that a central aspect of obesity might be an altered reward processing in the cortico-basal ganglia network similar to what has been found in models of addiction^10^. Thus, the controversial concept of obesity being a disorder based on food addiction has been postulated^11^. In line with this, obesity could be regarded as another neuropsychiatric brain circuit disorder similar to addiction, obsessive-compulsive disorder or Parkinson’s disease, which is supported by recent progress in the molecular and optogenetic dissection of the neurocircuitry involved in the regulation of food intake and metabolism^12^. The etiologies of circuit dysfunction are multivarious including damage of specific neuronal pathways, loss of individual neuronal populations and aberrant functional activity characterized by pathological oscillatory activity^13^. While a number of these aspects like impaired dopaminergic neurotransmission^14^ or neuronal injury due to hypothalamic inflammation^15^ have been addressed in the context of obesity, the question of whether aberrant network activity occurs in the involved neuronal circuits has not been clarified. To address this important question we aimed to characterize information processing in the limbic cortico-basal ganglia loop by simultaneous multi-site local field potential (LFP) recordings. LFPs are extracellular signals that are primarily generated by the summed activity of excitatory and inhibitory synaptic potentials within groups of neurons^16^. Due to their biophysical properties LFPs pose an opportunity to characterize the coordination of neuronal population activity within a defined network^16^. Recent cumulative evidence suggests that oscillations are of paramount importance for interneuronal communication in small- and large-scale neuronal networks^17,18^ with oscillations at different frequencies subserving different functions^17^. Furthermore the coherence of oscillatory signals between different brain areas has been proposed as a marker of functional connectivity that regulates neuronal communication through selective synchronization of neuronal populations^19^. The term oscillatory signaling as it is used here refers to the general description of the power and coherence of oscillatory LFPs within and between specific neuronal target areas. Recent studies on human patients suffering from debilitating neuropsychiatric diseases such as Parkinson´s disease, obsessive-compulsive disorder and depression have demonstrated a close relation between altered oscillations at specific frequencies and disease symptoms, suggesting a causal relation^20–22^. In the current study, we performed parallel *in vivo*-electrophysiological recordings of LFPs under urethane anesthesia from the medial prefrontal cortex (mPFC), nucleus accumbens (NAC) and the ventral tegmental area (VTA) at baseline and during a standardized intraperitoneal glucose tolerance test. We demonstrate that HFD induced obesity is characterized by an increase of beta oscillatory power in the limbic cortico-basal ganglia loop that is most pronounced in the NAC and accompanied by a decreased coherence in the beta frequency band between the NAC and the mPFC. NAC beta activity is highly correlated to diet-induced elevations of insulin and leptin levels at baseline while showing a differential group-dependent response towards a systemic glucose challenge.

## Material and Methods

### Animal model

In total 75 male wistar rats (RccHanWist, Harlan Laboratories, Netherlands) aged 14–16 weeks were used for this experiment. Rats were housed in a temperature- and humidity-controlled environment with 12-h light dark cycle with food and water available ad libitum. Rats were housed in groups of up to 4 animals. We opted for group housing, because a lower response for diet induced obesity has been shown for single housed animals^23^. In the first experimental set 45 rats were divided in two groups (control group N = 15, HFD group N = 30) and fed over a period of four weeks. There were no significant weight differences between the groups before the start of the experiments. Animals were randomly assigned to the groups. The control group received a standard lab chow diet (TD.2014, Harlan Laboratories, Netherlands). 45 animals were fed a high fat diet (HFD) with 45% kcal from fat (TD08811, Harlan Laboratories, Netherlands) for two weeks. All animals were ranked according to their body weight. The 15 animals with the lowest and highest body weight gain after the first two weeks were selected (mean body weight 374 vs. 340 g), further subclassified as HFD high response (HFD-H, N = 15) or HFD low response group (HFD-L, N = 15) and fed for two additional weeks. The initial intent was to select two metabolically distinct groups as it has been described before^24^. In our experiment, we could not replicate this phenotype after four weeks of HFD feeding. As shown in the Supplementary Material (Table S1, Figure S2) we have a rather broad distribution with respect to the extent of the metabolic phenotype. We therefore did not sub specify the groups for the further analysis. In the first cohort, 13 of 45 animals were excluded from final analysis (Control N = 12, HFD N = 20) for the following reasons: no AS detectable (N = 5), death during procedure (N = 4), incorrect glucose injection (N = 2), electrode malfunction (N = 1), no sufficient blood sampling (N = 1). In a second cohort 30 male wistar rats aged 14–16 weeks received an intracerebroventricular insulin (N = 15) or saline (N = 15) injection. In this cohort, we excluded 7 of 30 animals from the final analysis (insulin N = 12, saline N = 11) for the following reasons: death during procedure (N = 3), no AS detectable (N = 3), electrode malfunction (N = 1).The study was conducted in accordance with German Animal Welfare Act (last revised in 2014) and European regulations (2013/63/EU). All experiments were approved in advance by the local animal welfare authority (LaGeSo, Berlin) and conformed to local department and international guidelines.

### *In vivo* electrophysiology

*In vivo* electrophysiological recordings were made as previously described^25^. In brief, anesthesia was induced and maintained with urethane (1.3 g/kg i.p., Sigma–Aldrich, Germany). Rats were placed in a stereotactic frame (David Kopf Instruments, CA, USA) with heads fixed with atraumatic ear bars. Ophthalmic ointment (Bepanthen™, Bayer, Germany) was applied to prevent corneal dehydration. Body temperature of 37 ± 0.5 °C was maintained throughout surgery using a self-adjusting heating pad (CMA, Sweden). After incision of the skin, the head was aligned to the flat skull position using the rat alignment tool (David Kopf Instruments, CA, USA). Three customized parylene insulated tungsten electrodes (Microprobes, USA, tip diameter 4 µm, impedance 1MOhm) were implanted unilaterally into the left medial prefrontal cortex (AP = +3.2, ML = +0.7, DV = −4.0), nucleus accumbens shell (AP = +1.8, ML = +0.7, DV = −7.0) and the ventral tegmental area (AP = −5.0, ML = +0.7, DV = −8.3). All coordinates are given in mm in reference to Bregma^26^. All electrodes were secured with acrylic dental cement (Technovit®, Heraeus-Kulzer, Germany). Animals and implanted electrodes were left fixed to the stereotactic frame throughout the experiment. Recording electrodes were referenced against a custom-made Ag-AgCl electrode that was positioned in the epidural space above the ipislateral cerebellum. Signals were bandpass filtered (0.05 Hz–300 Hz), amplified, sampled at 1 kHz and digitized using a programmable neuronal data acquisition system (Omniplex, Plexon, Texas, USA) and stored for later offline data analysis.

### Data analysis

LFP recording under urethane-anesthesia was used as a well-established model for investigating basal ganglia activity and functional connectivity of the cortico-striatal system^27,28^. We focused our analysis on activated states (AS) that are characterized by fast, low amplitude signals that have been found to resemble oscillatory activity in the awake state independent of movement artifacts and motivational states^27,29,30^. 50-second periods of an AS was selected visually as described previously^25,31^ (for more detailed description and representative depiction of the raw signal see supplementary material). The same time segments were also used for analysis of LFPs from the NAC and the VTA. Power spectral densities of the LFP data segments were calculated by employing the Fast Fourier Transform function. The frequency spectrum was divided into standard EEG bands^32^. Power spectra were normalized to total power between 7 and 100 Hz and further expressed in arbitrary units (a.u.). Peak oscillatory activity was determined by selecting the peak frequency bin with the highest power value compared to its preceding frequency bin within the 13–20 Hz frame of the low beta frequency band. NAC beta peak frequency power was calculated as mean power of this and the two adjacent frequency bins. Averaged power across the specific frequency bands was used for statistical comparisons. To measure functional connectivity between the three different target areas we performed a coherence analysis^33^. Coherency is defined as the normalized cross-spectrum. Coherence is defined as the absolute value of coherency reflecting the linear relationship between the phase consistency of two signals at a specific frequency^34^. To exclude volume conduction as a confounder we focused on the calculation of the imaginary part of coherence (iCoherence; out of phase part of coherence^34^. For the coherence analysis the same time fragments as for the power spectrum density calculations were used^35^. In the first cohort, three separate time frames were analyzed: one during baseline recording and two during the performance of the i.p. glucose tolerance test (one and two hours after the glucose injection). In the second cohort, two time frames one before and one after intracerebroventricular injection of insulin or saline were analyzed. Correct electrode tip placements were histologically verified using sections stained with standard Nissl (for further description of data analysis and histology protocols see supplementary material).

### i.p. Glucose tolerance test, i.c.v. insulin injection and hormone measurements

Standard i.p. glucose tolerance testing was performed using a 20% glucose solution in a weight-adjusted dosage (2 mg/g body weight). The procedure started 7–8 hours after food removal and initiating of the anesthesia. Whole tail blood sampling was performed at 0, 30, 60, 90 and 120 minutes. Glucose levels were measured using a standard measurement devise (Contour XT, Bayer, Germany). Insulin and leptin measurements were performed using commercially available ELISA kits (10-1247-01, Mercodia, Sweden, Cat.Nr. MOB00, R&D Systems, USA). For further information, see supplementary material. The intracerebroventricular insulin injection was performed using a 33-gauge blunt-tip cannula fixed on a 10 μl Hamilton syringe (WorldPrecision Instruments, FL, USA). The third ventricle was targeted using the stereotactic coordinates AP: −2.3, ML: 0.0, DV: −8.5 mm. 2 μl of 4mU/l insulin or saline was injected at a pace of 1 μl/min via a precision syringe pump (Micro4™, World Precision Instruments, FL, USA).

### Data availability statement

The datasets generated during and analyzed during the current study are available from the corresponding author on reasonable request.

### Statistics

Statistical calculations were performed using SPSS 20.0 (IBM Statistics, Germany). All values are presented as mean and standard error of the mean (SEM). Metabolic parameters were log transformed. Normal distribution was tested using Shapiro Wilk test. Normally distributed metabolic parameters and electrophysiological data were analyzed using student’s t-test. Skewed variables (body weight) were analyzed using Mann-Whitney-U-test. For subgroup analysis one-way ANOVA and Kruskall-Wallis test were used accordingly. Bonferroni correction was applied to adjust for multiple comparisons. To test for a group time interaction a split-plot repeated measures ANOVA was performed. Repeated-measures ANOVA was used to analyze differences in beta activity between the different metabolic conditions. Correlations were calculated by Spearman’s rank correlation coefficient. We used a linear regression model to adjust for cortical slow wave activity, time point of sampling and body weight for evaluating associations between oscillatory activity and insulin levels. The significance level was chosen as *p* < 0.05.

## Results

### Metabolic baseline characteristics

In the main cohort, rats were randomly assigned to receive either a normal chow (controls) or a high fat diet (HFD) for a period of 4 weeks. The HFD group reached a significantly higher body weight than the age-matched control animals (Table 1). Baseline glucose values showed no significant difference between the two groups (Table 1). The standard i.p. glucose tolerance test (GTT) was conducted during the electrophysiological recordings under urethane anesthesia revealing a significantly higher area under the curve (AUC) glucose level for the HFD group compared to controls. In accordance, insulin levels at baseline as well as the insulin AUC were higher in HFD animals (Table 1). Basal serum leptin concentrations were more than doubled in the HFD group compared to controls (Table 1). The time course of glucose and insulin levels during the i.p. GTT are presented in the supplementary material (Figure S2). Of note are the overall increased serum concentrations and rather constant elevation over the 2-hour test duration that are known to manifest under general anesthesia with urethane.

**Table 1:** Baseline metabolic characteristics of the groups: values are depicted as mean value (S.E.M.) for normal chow group (Control N = 12), high fat diet group (HFD N = 20).

	Control	HFD
Body Weight [g]	401.1 (3.5)	457.7 (11.9)**
Baseline Glucose [mg/dl]	132.7 (8.3)	157.6 (8.4)
AUC Glucose [a.u.]	29065.0 (2388.3)	36414.8 (1940.1)*
Baseline Insulin [µg/l]	3.7 (0.4)	8.3(0.6)**
AUC Insulin [a.u.]	663.0 (47.1)	1391.3 (90.2)**
Leptin [ng/ml]	11.0 (2.44)	25.0 (2.7)*

T-test was used to compare metabolic parameters, *p < 0.05, **p < 0.001.

The further analysis of the sub classification within the HFD group based on a rather high versus low initial weight gain under high fat diet exposure (HFD-H vs. HFD-L) revealed no significant differences between the subgroups with respect to body weight, glucose and leptin concentrations. Solely insulin concentrations and insulin AUC separated these two subgroups (Supplementary Material Table S1). The metabolic characterization indicated a distinct between group difference for animals receiving normal chow versus receiving a high fat diet. The metabolic phenotype within the HFD group on the other hand displays a rather continuous spread (Supplementary Material Figure S2). We therefore focused for the primary analysis on the comparison between animals receiving either control chow or a high fat diet. In the first cohort, 32 of the 45 animals were included in the analysis. 13 animals were excluded for the following reasons: Control group - no AS detectable (N = 1), no raise in glucose levels presumably due to incorrect glucose injection (N = 2); HFD-H group - no AS detectable (N = 2), no sufficient blood sampling (N = 1), death during procedure (N = 2); HFD-L group - no AS detectable (N = 2), electrode malfunction (N = 1), death during procedure (N = 2). In the second cohort for the icv insulin injection we included 23 of 30 animals. Three animals died during the procedure. In one case the electrode in the nucleus accumbens did not record properly. Three animals had to be excluded since no activated state could be detected.

### HFD group showed increased beta power in the cortico-basal ganglia loop at baseline

**Figure 1:**
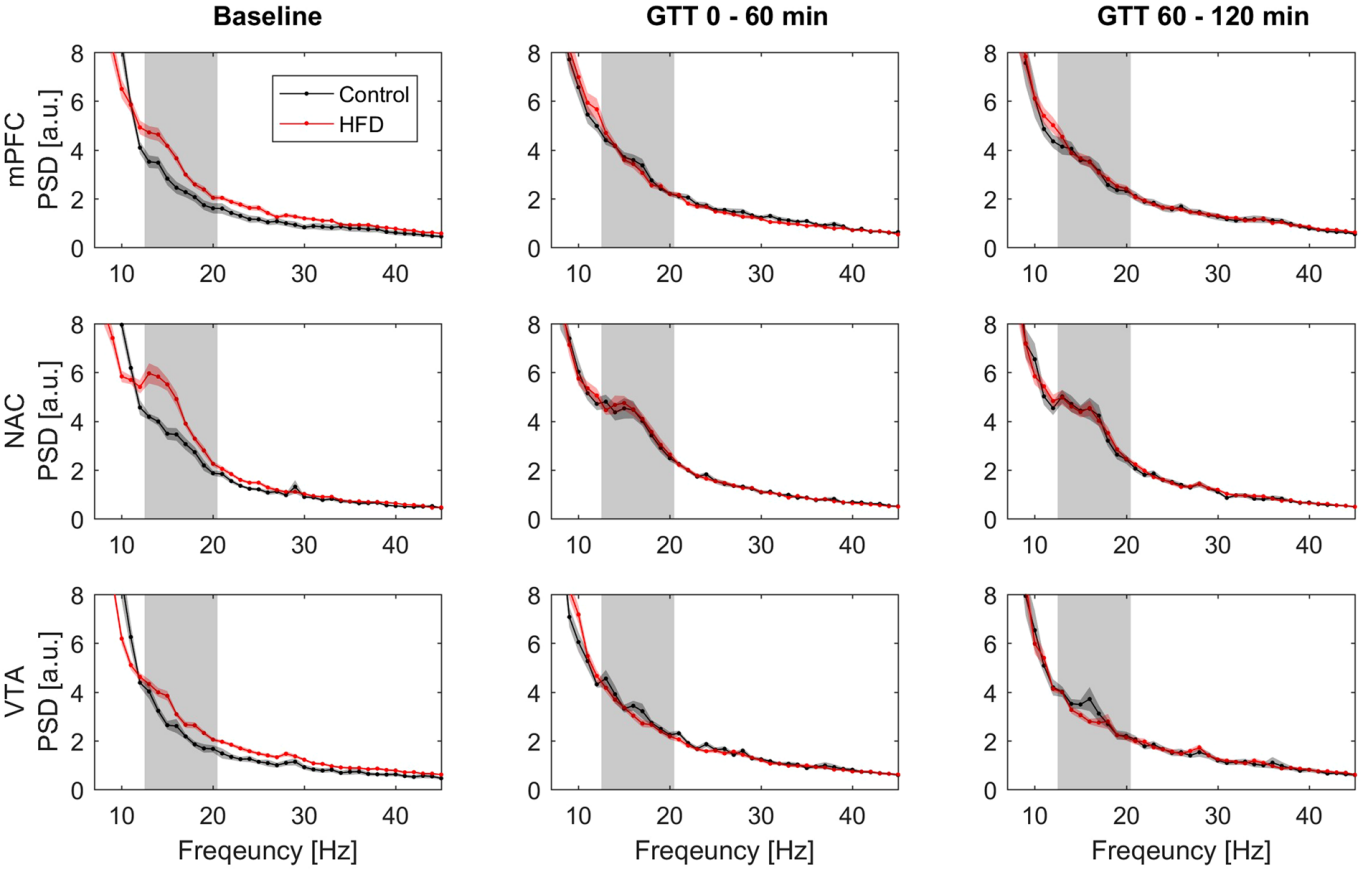
Power Spectral Density (PSD) analysis over a frequency spectrum from 7 to 45 Hz depicted as mean arbitrary unit [A.U.] for 1 Hz frequency bins ± SEM. Row one: medial prefrontal cortex (mPFC), row two: nucleus accumbens shell region (NAC) and row three: ventral tegmentum area (VTA). The left column shows PSD data for the baseline condition, measured before the initiation of the glucose tolerance test (Control N = 12, HFD N = 20). The middle column shows PSD data measured during the first hour of the glucose tolerance test (Control N = 11, HFD N = 18) and the right column during the second hour of the test (Control N = 11, HFD N = 18). Grey area marks the low beta frequency spectrum (13–20 Hz).

We performed simultaneous local field potential recordings from the mPFC, the VTA, and the NAC in all animals. Epochs of robust global cortical activation (activated states) were analyzed at baseline and during the first and second half of the glucose tolerance test. To exclude that urethane might have had a different effect on cortical synchronization states in animals with diverse body compositions we compared the power of low frequency oscillations (<1 Hz) of the chosen activated states between the different experimental groups. As urethane anesthesia is dominated by oscillations at this frequency band, a difference in anesthesia depth or of the frequency content of the activated states would be detectable by this analysis. We could not find any significant differences in this measurement (Table 2). The only definite frequency peak over the investigated frequency spectrum was identified in the low beta frequency band (13–20 Hz) in recordings from the NAC with a concordant but less pronounced effect in VTA and mPFC (Fig. 1). Calculation of the mean power of the frequency band revealed a significant increase in low beta power in animals fed a HFD (Table 2).

**Table 2:** Statistical analysis of mean power and iCoherence of the low beta frequency band under baseline condition.

Frequency Band		Mean Power [A.U.]	S.E.M.	p-value
*mPFC SlowWave 0-1* *Hz*	Control	157.2	51.0	0.387
	HFD	251.5	77.0	
*mPFC Beta 13–20* *Hz*	Control	2.2	0.2	<0.001
	HFD	**3.0**	0.1	
*NAC Beta 13–20* *Hz*	Control	2.8	0.2	<0.001
	HFD	**3.8**	0.1	
*VTA Beta 13–20* *Hz*	Control	2.2	0.1	<0.001
	HFD	**2.8**	0.1	
Frequency Band		Mean Coherence [A.U.]	S.E.M.	p-value
*iCOH mPFC NAC Beta 13–20* *Hz*	Control	0.20	0.03	0.05
	HFD	**0.13**	0.01	
*iCOH mPFC VTA Beta 13–20* *Hz*	Control	0.25	0.03	0.01
	HFD	0.15	0.01	
*iCOH VTA NAC Beta 13–20* *Hz*	Control	0.11	0.01	0.28
	HFD	0.13	0.01	

Cortical slow wave activity (mPFC SlowWave 0–1 Hz) was analyzed as control parameter for a potential overall influence of anesthesia. Visually identified between group difference (Control N = 12, HFD N = 20) in PSD and iCoherence was analyzed using student’s t-test.

### HFD group showed a selective decrease of cortico-subcortical coherence in the beta frequency band at baseline

**Figure 2:**
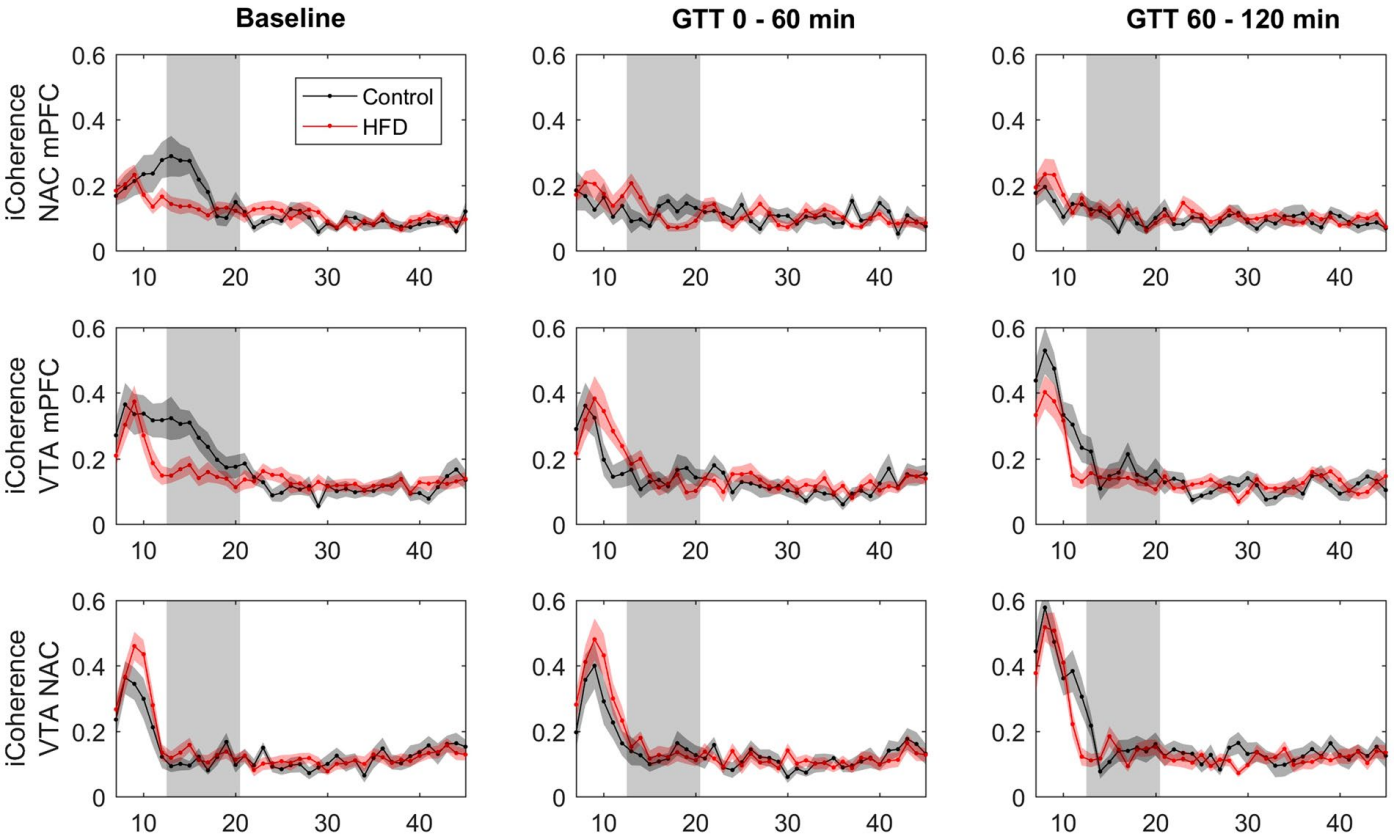
iCoherence analysis over a frequency spectrum from 7 to 45 Hz depicted as mean coherence for 1 Hz frequency bins ± SEM. Row one: medial prefrontal cortex (mPFC) with nucleus accumbens shell region (NAC), row two: medial prefrontal cortex (mPFC) with ventral tegmentum area (VTA) and row three: nucleus accumbens shell region (NAC) with ventral tegmentum area (VTA). The left column shows PSD data for the baseline condition, measured before the initiation of the glucose tolerance test (Control N = 12, HFD N = 20). The middle column shows PSD data measured during the first hour of the glucose tolerance test (Control N = 11, HFD N = 18) and the right column during the second hour of the test (Control N = 11, HFD N = 18). Grey area marks the low beta frequency spectrum from 13 to 20 Hz.

To analyze the functional connectivity within the limbic loop of the cortex-basal ganglia network we analyzed iCoherency between the recorded cortical and subcortical targets (Fig. 2). At baseline iCoherence values calculated for the combination of mPFC and the NAC as well as for the mPFC and the VTA showed a significantly lower mean coherence in the low beta frequency band in animals fed with a high fat diet (Table 2). No consistent difference was detected for the iCoherence between VTA and NAC.

### In hyperglycemia beta oscillations increased and beta coherence decreased in control animals while remaining stable in the HFD group

**Figure 3:**
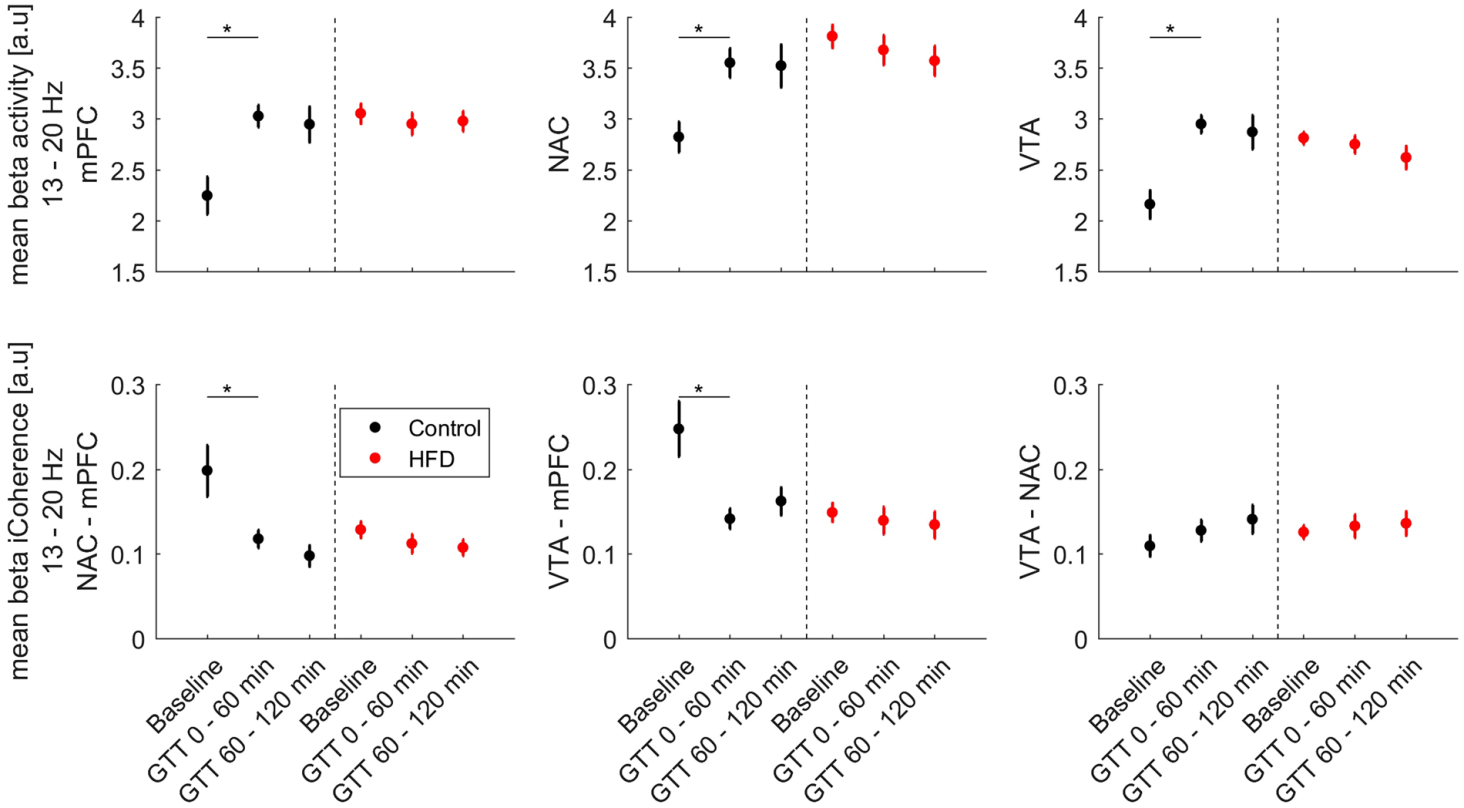
Mean low beta power and low beta-iCoherence (13–20 Hz) ± SEM for the three measured AS conditions: Baseline (Control N = 12, HFD N = 20), 1^st^ (Control N = 11, HFD N = 18) and 2^nd^ (Control N = 11, HFD N = 18) time segment after i.p.-glucose injection. Graphs depict the three targeted areas medial prefrontal cortex (mPFC), nucleus accumbens (NAC) and ventral tegmentum area (VTA). Paired t-test was used for post-hoc testing to analyze differences in beta activity between the conditions, *p < 0.05.

After the completion of the baseline recordings we proceeded with LFP recordings after an i.p. injection of 2 mg/g body weight of glucose. Power spectral densities were calculated after the first and the second hour following the injection (Fig. 1). The power analysis of the first time segment showed a significantly increased low beta power in control animals (Figs 1 and 3), that was most pronounced in the NAC. In the high fat diet group, low beta power remained on the elevated level observed before the glucose challenge (Fig. 3). This effect remained stable for the second time point. Repeated-measure ANOVA analysis revealed a significant time group interaction (Tests of Within-Subjects Contrasts, Mean Square 3.91, p < 0.001) for the comparison of NAC beta activity in the three measured AS timeframes. Beta power levels were not statistically different between control and HFD-animals in hyperglycemia at both time points (Fig. 3). Beta coherence between NAC and mPFC displayed a similar pattern with opposite direction (Fig. 2). The initially higher beta coherence in control animals was significantly reduced after glucose application while similar modulation in the HFD group (time group interaction - Tests of Within-Subjects Contrasts, Mean Square 0.32, p = 0.007).

### NAC beta oscillatory power correlated with metabolic markers at baseline

**Figure 4:**
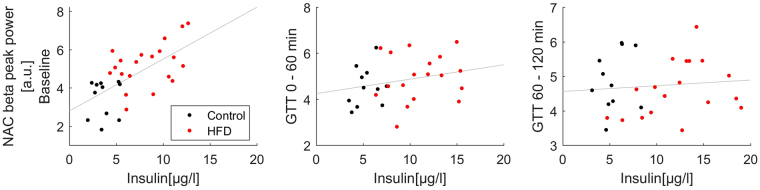
Correlation of NAC beta peak power and insulin levels measured before (N = 31) and during the first (N = 29) and second half (N = 29) of the glucose tolerance test.

To further evaluate possible interactions between oscillatory phenomena in the limbic cortico-striatal system and the metabolic profile in general, we analyzed correlations of baseline NAC mean beta activity (13–20 Hz) and NAC beta peak frequency power with the recorded metabolic markers of the whole animal cohort independent of group assignment (Supplementary Material Table S2). Bivariate correlation analysis revealed strong positive associations between NAC beta peak power with body weight (r = 0.49, p = 0.004), glucose AUC (r = 0.46, p = 0.008) and serum leptin levels (r = 0.49, p = 0.005; scatter plots provided in Supplementary Material Figure S3). The strongest positive correlation consisted between NAC beta peak power and baseline insulin levels (Fig. 4). In the linear regression analysis this association (adj. r² = 0.44, ß = 0.68, p < 0.001) persisted even when correcting the model for slow wave activity, measurement time point and the animal’s body weight (adj. r² = 0.39, ß = 0.59, p < 0.013). Analysis of the relationship between NAC beta peak power and baseline insulin within the two experimental groups revealed a significant association within the HFD group (r = 0.47, p = 0.037) but not in control animals (r = 0.16, p = 0.65). This association was specific only for NAC beta power. It was not detected in mPFC and VTA nor did beta coherence correlate with markers of the metabolic phenotype. The baseline association of insulin and NAC beta peak power disappeared after i.p. glucose application and was not detected anymore during the glucose tolerance test (Fig. 4).

### ICV insulin injection increases NAC beta oscillatory power

**Figure 5:**
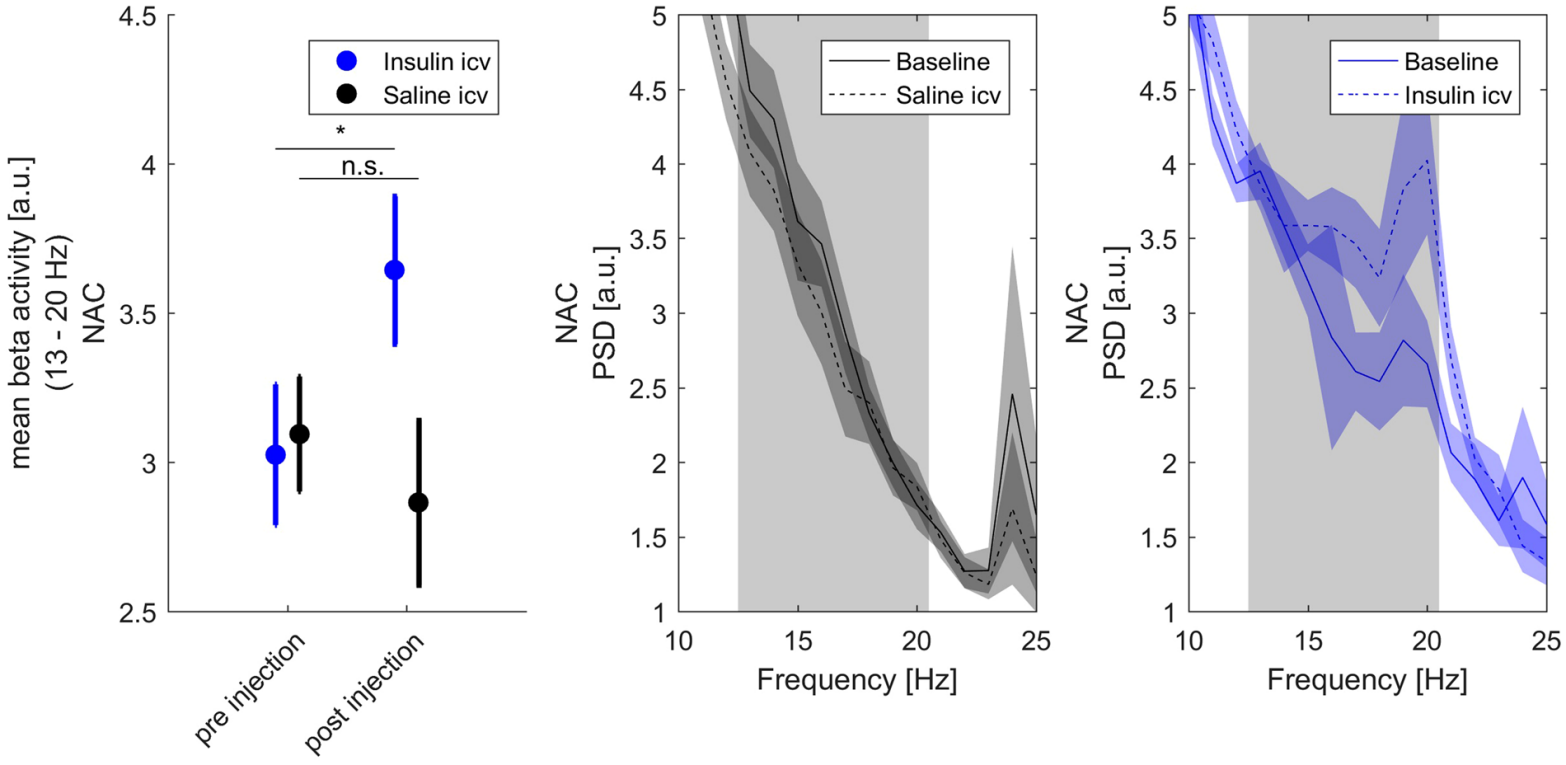
Mean low beta power and corresponding power spectra in the NAC of lean animals measured before and after microinjection of either insulin (N = 12) or saline (N = 11) into the third ventricle. Paired t-test was used for post-hoc testing to analyze differences in beta activity between conditions, *p < 0.05.

To investigate if the observed increase in beta oscillatory activity in the NAC was resulting from central insulin signaling, we performed an additional set of experiments in lean control animals. Mean beta oscillatory power in the low beta frequency band of the NAC was measured before and after central insulin injection (Fig. 5). ICV insulin application induced a significant increase in mean NAC beta power (pre injection 3.03 [a.u.], post injection 3.64 [a.u.], p = 0.031) while sham injection induced no change (pre injection 3.10, post injection 2.87, p = 0.279). Of note the induced beta peak had as maximum around at 19–20 Hz, while the observed beta peak after systemic glucose application spiked at around 15 Hz. There were no significant between group differences with respect to body weight (insulin group 387.1 g vs. sham group 384.9 g) and time between AS and insulin application (pre injection: insulin group −21.0 min vs. sham group −19.5 min, p = 0.883; post injection: insulin group +33.4 min vs. sham group post injection +42.8 min, p = 0.303).

## Discussion

In the current study we followed a systematic combined approach of endocrinological testing and characterization of the neuronal network activity in a rat model of obesity. Based on the idea of a circuit disorder with abnormal reward processing in diet induced obesity we explored oscillatory LFP activity within the limbic circuit of the cortico-basal ganglia loop before and during a systemic glucose challenge. Our main findings are: 1.) A distinct peak in the low beta frequency band was found in the nucleus accumbens of the animals that received a high fat diet for 4 weeks. 2.) Beta power correlated with body weight and serum leptin as well as insulin levels at baseline pointing to an association between central beta activity and peripheral metabolic parameters. 3.) Beta power increased in controls during a defined i.p.-glucose challenge but no further increase occurred in animals pretreated with a high fat diet. 4.) Cortico-subcortical beta band coherence between the mPFC and the NAC as well as the mPFC and the VTA was reduced in the HFD groups and was differentially modulated after the systemic glucose application. Oscillatory brain activity has only rarely been studied in the context of obesity. In a magnetoencephalographic (MEG) study with obese and lean humans it could be demonstrated that cortical beta activity can be increased by insulin in lean subjects but not in obese patients^36^. This study is partly in line with what we could show in our data set and underscores the hypothesis that insulin might play an important role in the context of hormonal and possibly disease related modulation of brain rhythms. In a recent EEG study elevated cortical beta activity was identified in overweight women depicting a linear relationship between relative beta band power and body fat mass^37^. While MEG/EEG-based approaches are limited to the analysis of cortical activity, invasive recording approaches allow the assessment of subcortical components of the cortico-striatal system. Utilizing this invasive approach gamma oscillations have been identified to organize top-down signaling to the hypothalamus enabling food seeking behavior in mice^38^.

Our data show a more pronounced alteration of oscillatory activity in the NAC compared to mPFC suggesting a pivotal role of subcortical areas for the generation of oscillatory signaling. Based on the well investigated role of insulin as a brain satiety signal^39^ interfering with dopaminergic neurotransmission^40,41^ and the discovery of exaggerated beta oscillatory activity in the dopamine depleted basal ganglia system of parkinsonian patients^20^, it is tempting to formulate the hypothesis that beta oscillatory activity and coherence in obesity might constitute a relevant modality of reward regulation through central insulin signaling. To investigate the potential role of brain insulin signaling in this context, we performed an additional set of experiments evaluating the change of NAC beta oscillatory power following a direct insulin injection in the third ventricle. This approach of delivering insulin directly into the brain has been used in a number of animal studies to explore central insulin action as a satiety signal in discrimination to its glucose lowering effect in the periphery^39^. While the potential of brain insulin signaling to lower food intake is well established, in a recent animal study it has been demonstrated that the action of appetite suppressant drugs is mediated by dopaminergic signaling and associated with changes in beta, theta and delta band LFP oscillations^42^. The results of our experiments show that central insulin application induced an increase in NAC beta oscillations. The frequency peaks of this increased beta oscillatory activity vary between systemic glucose and i.c.v. insulin application, potentially indication that insulin is relevant but not the only factor mediating this effect. Since we are at this point unable to address the question of what increased beta oscillatory signaling means for neuronal computation in obesity, it is interesting to consider what is known from other contexts. Data from animal and human studies in various fields led to the formulation of the hypothesis that beta band activity has a primary role in health and disease. On the one hand, under physiological conditions beta oscillations seem to promote the maintenance of the current sensorimotor or cognitive state^43^. On the other hand increased beta activity is associated with a pathological persistence of the current sensorimotor state, which might directly result in the disease symptoms in PD that are characterized by a lack of movement and problems in initiating movements^20^. Since these investigations on oscillatory activity have given rise to a number of promising novel neuromodulatory approaches like closed-loop deep brain stimulation^44^ or patterned transcranial stimulation^45^, a further characterization of oscillatory network activity could help to facilitate the development of novel therapies for obesity. In fact preclinical and clinical studies on DBS in obesity have brought first evidence that this therapy might be effective^46^.

Our experimental design comes with a number of relevant limitations. A drawback is that we have conducted our experiments under general anesthesia with urethane. Urethane like almost all general anesthetics has been described to have an impact on various hormone levels especially with respect to the glucose metabolism. Systemic administration of urethane leads to a sympathetic response with release of insulin antagonistic hormones inducing a raise of glucose values and an attenuation of insulin’s capacity to lower serum glucose levels^47,48^. These effects can clearly be recognized in the metabolic characterization of the animals investigated in our experiment (Table 1). While the rather constant increase in blood glucose and insulin levels after the systemic challenge facilitates the analysis of oscillatory activity with respect to metabolic parameter, it exemplifies the non-physiologic condition of the used approach. Nevertheless, the relative differences between the groups match the expected metabolic changes following a 4 week high fat diet feeding in terms of an up to 20% increase in body weight, accompanied by an impaired glucose tolerance in the GTT as measured by AUC glucose and insulin levels without manifestation of a different baseline glucose level. Despite that, the usage of urethane anesthesia has a number of essential advantages for testing the hypothesis of a potential neuro-endocrine interaction. It produces a stable and long lasting anesthesia that allows performing electrophysiological experiments for hours^29^. It is therefore possible to record and analyze signals that are not compromised by sensory stimuli (e.g. pain and stress during blood sampling) and specific behaviors with respect to metabolic alterations. The two very well defined cortical activation states - slow wave activity (SWA) and activated state (AS) - occur spontaneously under urethane anesthesia. While the SWA state resembles cortical activity during natural sleep, the AS shows many characteristics of brain activity patterns that can be found in awake and attentive individuals^49,50^. Admittedly, as an AS might resemble some characteristics of the awake state, it is not identical to it and specific neuronal responses can substantial vary when comparing urethane versus awake recordings^51^. Nevertheless the careful analysis of thesetwo stereotypic cortical activation states - slow wave activity (SWA) and activated state (AS) - secures a high level of comparability between recordings in different animals and therefore diminishes interindividual variations that can lower the sensitivity for the detection of differences between experimental groups. Urethane leaves neurotransmission in subcortical areas and in the peripheral nervous system relatively unaffected, which is why it is a suitable anesthetic to study basal ganglia function and cortico-basal ganglia interactions^52^. The electrophysiological phenotypes that can be detected under urethane anesthesia have been reproduced in awake recordings in numerous cases making the approach a widely used method for preclinical research despite the limitations associated with the application of general anesthesia^53–55^. In this regard, it is important to note that we have found no indication of an overall difference in oscillatory brain activity under urethane anesthesia between the groups of animals. By comparing the amplitude of low frequency oscillations (0–2 Hz), that signals the depth of the anesthesia, we can exclude that this might be an alternative explanation for the observed phenotype. Accordingly, even when adjusting for frontal slow wave activity as well as the animal’s body weight and the measurement time the highly significant association between baseline insulin levels and baseline NAC beta peak power persisted in a linear regression model. Another limitation of the present study is that we were not able to quantify cerebral insulin levels, which might be of importance to differentiate its effect on beta activity especially against the background of the potential relation between transport of insulin into the CSF to peripheral insulin sensitivity^56^. While beta oscillatory activity and coherence clearly differentiate between the two groups our investigation does not allow to clarify if obesity itself, high fat diet exposure or insulin resistance are the main drives behind the observed different electrophysiological patterns. In summary, the exploration of metabolic processes under a general urethane anesthesia comes with a number of limitations but it might offer the specific opportunity to explore an interaction between endocrine parameters and default-state network activity. We therefore propose the combined approach of endocrinological testing and analysis of default-state oscillatory signaling as novel complementary approach to explore neurohumoral interaction.

## Conclusion

In the present study, we are the first to identify differential beta oscillatory activity and coherence in the limbic cortico-basal ganglia loop in obesity that is strongly correlated with serum insulin and leptin levels. NAC beta oscillatory power is enhanced by hyperglycemia and can be induced by direct insulin application into the central nervous system. Our data support the hypothesis that obesity and insulin resistance may be considered as circuit disorders being characterized by dysfunctional oscillatory network activity in the limbic cortico-basal ganglia loop. Future studies should investigate the role of oscillatory brain activity in the pathophysiology of obesity and insulin resistance to enable the development of specific neuromodulatory therapies.

### Disclosure

Andrea Kühn|Consultancies: Medtronic, St. Jude Medical, Boston Scientific. Advisory Boards: Boston Scientific, Medtronic.

## Electronic supplementary material

Supplementary material
