# Supplementary material for: High-fat diet-induced obesity and insulin resistance are characterized by differential beta oscillatory signaling of the limbic cortico-basal ganglia loop
Source: Sci Rep. 2017 Nov 14;7:15555. doi: 10.1038/s41598-017-15872-x (PMC5686216; doi:10.1038/s41598-017-15872-x)
Supplement: Supplementary file 1 — Supplementary material [file 41598_2017_15872_MOESM1_ESM.pdf]

Supplementary material

High-fat diet-induced obesity and insulin resistance are characterized by differential beta oscillatory signaling of the limbic cortico-basal ganglia loop

Lukas Maurer*, Hui Tang*, Jens K. Haumesser, Jennifer Altschüler, Andrea A. Kühn, Joachim Spranger* and Christoph van Riesen*

Data Analysis

In urethane anesthesia two well defined cortical synchronization states have been described: the slow wave activity state (SWA) and the activated state (AS) [1]. The SWA state LFP signal is dominated by a slow oscillation of around 1Hz and has been shown to resemble activity patterns found in natural sleep. The activated state shows a much faster signal and has been found to be similar to LFPs from awake subjects [2]. The visual detection of slow wave activity and activated states is a standard procedure because these cortical synchronisation states are very easy to identify visually because of the characteristic differences in amplitude and rhythm. A representative example for the raw signal trace of a SWA and an AS period as shown in figure S1. Although those brain states may differ from neural activity seen in the unanesthetized brain, the urethane-anesthetized animal still serves as well-established model for characterizing basal ganglia activity [3,4] and functional connectivity within and between basal ganglia and cerebral cortex [2]. For our analysis, we focused on the activated state. LFPs recorded in the mPFC were used to identify 50s episodes of activated states by visual selecting and reduction of slow wave activity (< 1Hz) to < 20% compared to preceding 50s SWA episode. Animals that did not display robust cortical activation signals were not included into the further data analysis (Control N = -1, HFD-R N =-2, HFD-N N=-2). The same time segments were also used for analysis of LFPs from the NAC and the VTA. Three separate time segments were analyzed one during baseline recording and two during the performance of the i.p. glucose tolerance test (one and two hours after the glucose

injection). We use the start time of the glucose tolerance test as the reference time point. During the preceding baseline recording the AS closest to this point that did not show signs of artifacts was chosen. The same method was used for the selection of AS during the glucose tolerance test with the time points of 60 and 120 min after the glucose injection as a reference. One AS per animal and time point was used for further analysis, which is standard procedure [5]. Power spectral densities of the LFP data segments were calculated by employing the Fast Fourier Transform function (Spike 2 Version 6 data analysis software; Hann Window (1024 ms), 0.9766 Hz resolution). The frequency spectrum was divided into standard EEG bands [6]: sub-delta/slow wave (< 1 Hz), delta (2–4 Hz), theta (5–7Hz), alpha (8–12 Hz), low beta (13–20 Hz), high beta (13–30 Hz), low gamma (31–45 Hz) and high gamma (60–100 Hz). Power spectra were normalized to total power between 7 and 100 Hz and further expressed in arbitrary units (a.u.). Power was averaged across the specific frequency bands for statistical comparisons. Additionally NAC beta peak frequency was determined by selecting the peak frequency bin with the highest power value compared to its preceding frequency bin within the 13-20 Hz frame of the low beta frequency band. NAC beta peak frequency power was calculated as mean power of this and the two adjacent frequency bins. Correct electrode tip placements were histologically verified using sections stained with standard Nissl.

Histology

After the completion of the recordings, animals were sacrificed and transcardially perfused with 250ml of 0.1M phosphate buffered saline (PBS, pH 7.4) followed by 250 ml ice cold 4 % paraformaldehyde in PBS (PFA) as previously published [7]. Brains were removed and post-fixed in PFA for 24 h, then immersed in sucrose solutions of ascending concentrations for cryoprotection before being frosted at -80 °C until sectioning. 40 µm thick coronal sections were prepared with a cryotome (-20°C, Leica, Germany). Relevant sections for electrode placement verification were mounted on glass slides and stained with cresyl violet (Nissl staining). Trajectories, target structures and neighbouring structures were verified using a light microscope (Leica, Germany).

i.p. Glucose tolerance test (GTT):

Animals were taken out of their home cages at 07:00 AM. No preceding overnight fasting was conducted, but animals were fasted until anesthesia depth stabilized and all electrodes were implanted, which took approximately 4 - 6 hours. Thus, baseline electrode recording (starting after > 1 hour stabilization phase) and subsequent glucose application was started in a fasting situation. No systematic differences were found between the groups of animals for the starting times of the recordings and the GTT (data not shown). After determination of baseline blood glucose levels, each animal received an intraperitoneal injection of a 20 % glucose solution (G-20 %, B. Braun, Germany) in a weight-adjusted dosage (2mg/g body weight). Blood glucose levels were measured over a period of 2 hours at defined time points (30, 60, 90 and 120 min) from whole tail blood with a standard glucose measurement devise (Contour XT, Bayer, Germany).

Hormone measurement:

Blood samples of 150 µl per time point were taken from the tail vein at 0, 30, 60 and 120 min during the GTT. Samples were immediately stored on ice. After the completion of the GTT the samples were centrifuged and the supernatant blood plasma was stored at -80°C. Insulin measurement was performed using a commercially available ELISA kit (10-1247-01, Mercodia, Sweden). Leptin measurements were performed using a commercially available ELISA kit (Cat.Nr. MOB00, R&D Systems, USA).

Table S1

	Control	HFD-H	HFD-L
**Body Weight [g]**	401.1 (3.5)	480.3 (16.2) **	435.1 (14.9)
**Baseline Glucose [mg/dl]**	132.7 (8.3)	160.5 (10.3)	154.7 (13.7)
**AUC Glucose [a.u.]**	29065.0 (2388.3)	39217.5 (3087.6) *	33612.0 (2147.8)
**Baseline Insulin [µg/l]**	3.7 (0.4)	9.9 (0.7) ** §	6.6 (0.7) ##
**AUC Insulin [a.u.]**	663.0 (47.1)	1634.8 (96.9) ** §	1147.7 (108.5) ##
**Leptin [ng/ml]**	11.0 (2.44)	24.3 (3.4) *	25.7 (4.2) #

Table S1 | Baseline metabolic characteristics of the groups: values are depicted as mean value (S.E.M.) for normal chow group (Control N = 12), high fat diet high response (HFD-H N = 10) and high fat diet low response group (HFD-L N = 10). One-way ANOVA and post-hoc testing with Bonferroni correction was used to test log transformed metabolic parameters, ***** p < 0.05 for Control vs. HFD-R, ****** p < 0.001 for Control vs. HFD-R, **#** p < 0.05 for Control vs. HFD-N, **##** p < 0.001 for Control vs. HFD-N, **§** p < 0.05 for HFD-R vs. HFD-N

Figure S1

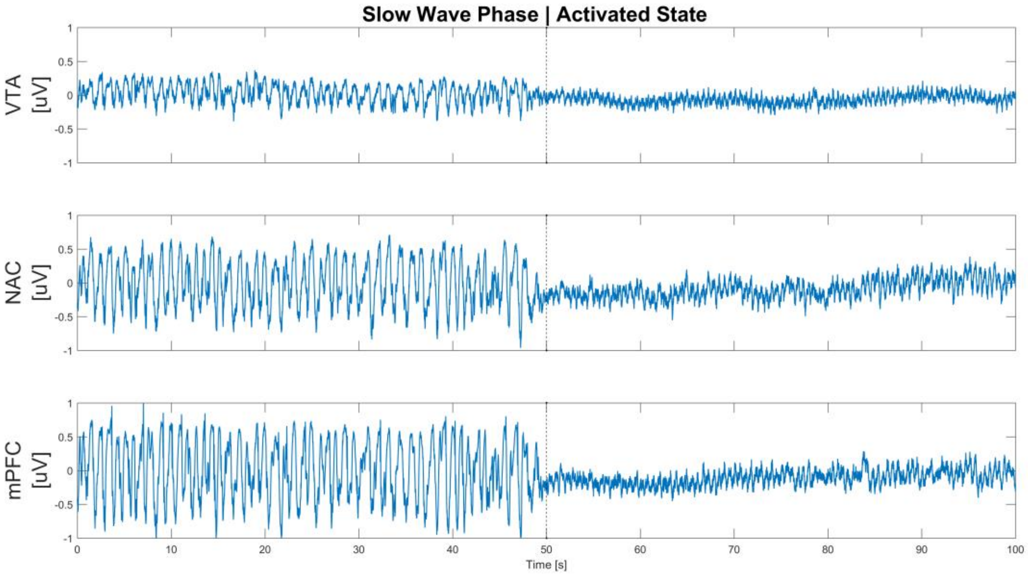

Figure S1 | Representative example of a raw trace of the recorded LFP signal for the three target areas medial prefrontal cortex (mPFC), nucleus accumbens (NAC) and ventral tegmentum area (VTA). The first 50 seconds period shows a slow wave activity phase that is followed by an activated state.

Figure S2

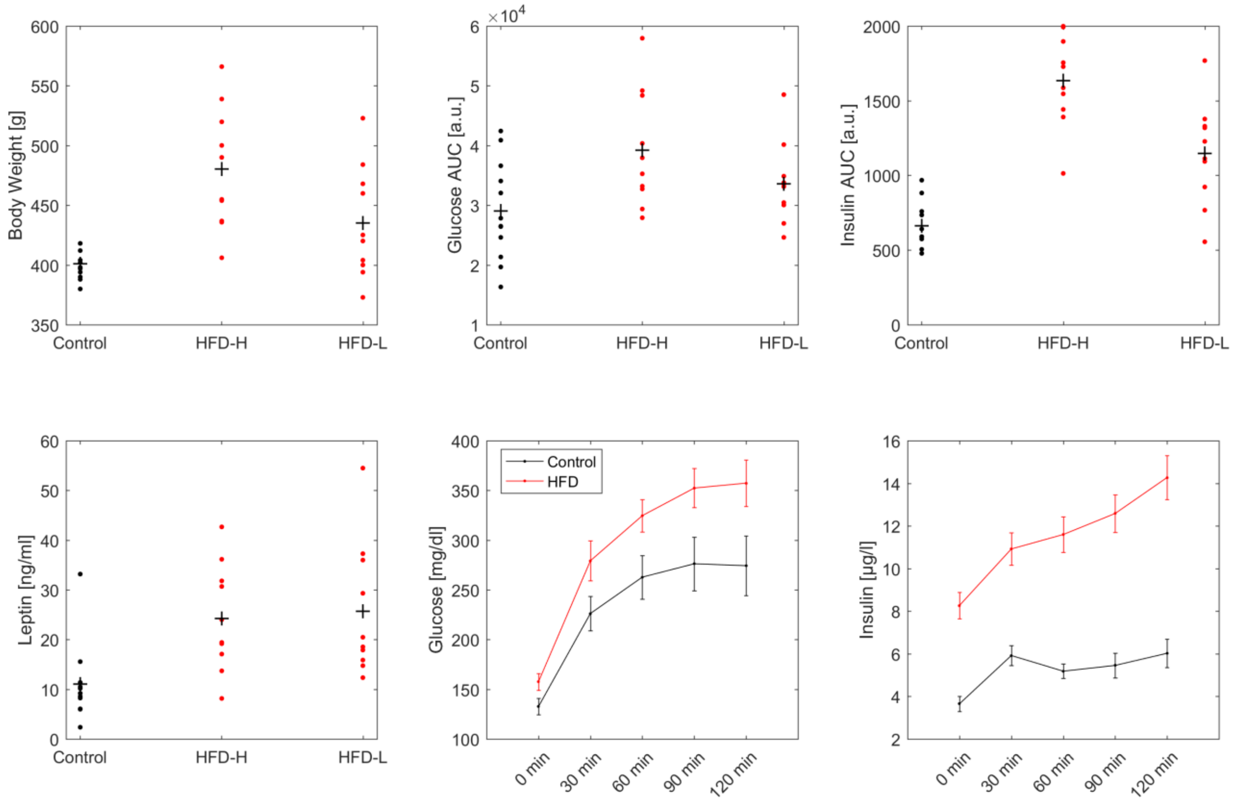

Figure S2 |Individual body weight, glucose AUC, insulin AUC and leptin of all animals included in the analysis (Control N = 12, HFD-H N = 10, HFD-L N = 10). i.p. GTT **-** Insulin and glucose levels measured every 30 minutes following i.p. injection of 20% glucose solution (2mg/g body weight) displayed as mean ± SEM for the control groups vs HFD.

Figure S3

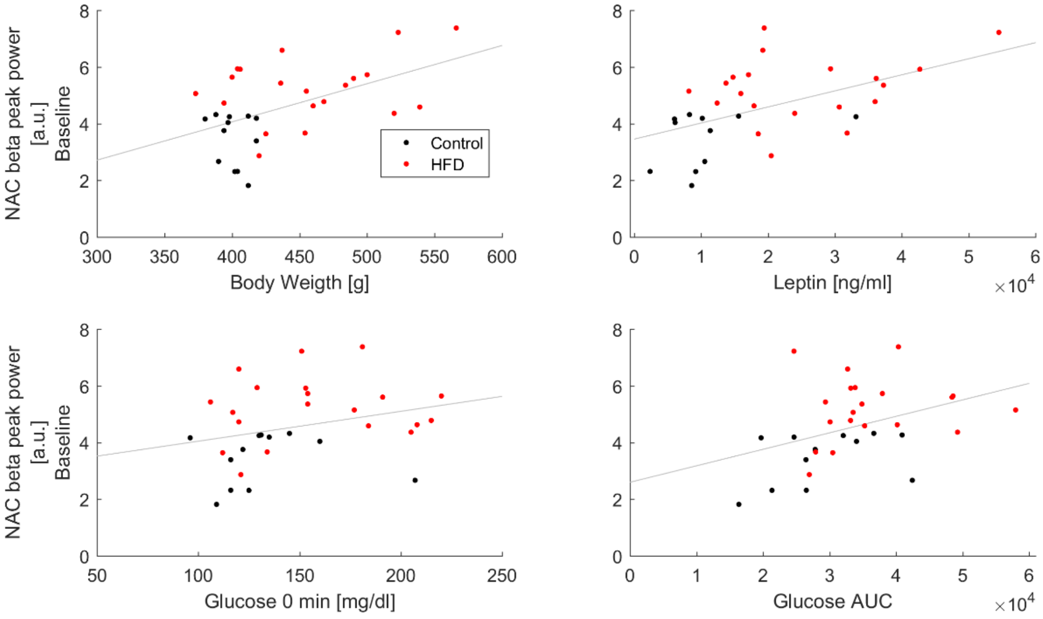

Figure S3 |Whole cohort bivariate correlations of mean baseline beta peak power within the beta frequency band of the nucleus accumbens with A: body weight (N = 32), B: serum leptin levels (N = 31), C: baseline glucose levels (N = 31) and D: subsequent glucose area under the curve during glucose tolerance test depicted as Spearman's rank correlation coefficient and corresponding p-value.

Table S2

Correlation Coefficient	Mean Power NAC Beta	Peak Power NAC Beta
Body Weight [g]	0.49*	0.49*
Baseline Glucose [mg/dl]	0.19	0.4*
Glucose 120 min [mg/dl]	0.34	0.50*
Glucose AUC	0.32	0.46*
Baseline Insulin [µg/l]	0.59***	0.65***
Insulin 120 min [µg/l]	0.63***	0.61***
Insulin AUC	0.53**	0.55**
Leptin [ng/ml]	0.41*	0.49**

Table S2 | Whole cohort bivariate correlations (N = 32) of mean baseline beta activity in the nucleus accumbens with metabolic parameters. Displayed for mean power in the low beta band (13-20Hz) and mean peak power (mean bin power of low beta peak frequency and the two adjacent frequency bins). Spearman's rank correlation coefficient displayed with ***** $p < 0.05$, ****** $p < 0.01$, ******* $p < 0.001$

References

1 Steriade, M. Corticothalamic resonance, states of vigilance and mentation. *Neuroscience* **101**, 243-276, doi:http://dx.doi.org/10.1016/S0306-4522(00)00353-5 (2000).

2 Magill, P. J. *et al.* Changes in functional connectivity within the rat striatopallidal axis during global brain activation in vivo. *J Neurosci* **26**, 6318-6329, doi:10.1523/JNEUROSCI.0620-06.2006 (2006).

3 Magill, P. J., Bolam, J. P. & Bevan, M. D. Dopamine regulates the impact of the cerebral cortex on the subthalamic nucleus-globus pallidus network. *Neuroscience* **106**, 313-330 (2001).

4 Mallet, N. *et al.* Disrupted dopamine transmission and the emergence of exaggerated beta oscillations in subthalamic nucleus and cerebral cortex. *J Neurosci* **28**, 4795-4806, doi:10.1523/JNEUROSCI.0123-08.2008 (2008).

5 Magill, P. J., Sharott, A., Bolam, J. P. & Brown, P. Brain State–Dependency of Coherent Oscillatory Activity in the Cerebral Cortex and Basal Ganglia of the Rat. *Journal of Neurophysiology* **92**, 2122-2136, doi:10.1152/jn.00333.2004 (2004).

6 Gazit, T. *et al.* Programmed deep brain stimulation synchronizes VTA gamma band field potential and alleviates depressive-like behavior in rats. *Neuropharmacology* **91**, 135-141, doi:10.1016/j.neuropharm.2014.12.003 (2015).

7 Beck, M. H. *et al.* Short- and long-term dopamine depletion causes enhanced beta oscillations in the cortico-basal ganglia loop of parkinsonian rats. *Experimental Neurology* **286**, 124-136, doi:http://dx.doi.org/10.1016/j.expneurol.2016.10.005 (2016).
